# Medical College of Georgia, Augusta

**Published:** 1872-08

**Authors:** 


Medical College of Georgia, Augusta.—We have received the forty-first annual announcement of the Medical College of Georgia, in the city of Augusta. In announcing the opening of their next course of instruction, the Trustees and Faculty congratulate themselves upon the results of unremitting endeavors of the Faculty to follow closely the great reform now progressing in medical science. They announce new and more complete arrangements for clinical instruction, and, we are pleased to see, have not fallen into the error of lowering the fees of the institution.

Among other pamphlets worthy of attention, we have received a paper from John T. Darby, M.D., Professor of Anatomy and Surgery in the University of South-Carolina, upon "Extra-Uterine Pregnancy," which will be noticed more particularly in the next number of the Journal.

Bellevue Hospital Medical College, New-York.—In our advertising department, will be found the announcement of this leading school of medicine. It needs no commendation from us.

